# CD4+ T Cell Regulatory Network Underlies the Decrease in Th1 and the Increase in Anergic and Th17 Subsets in Severe COVID-19

**DOI:** 10.3390/pathogens12010018

**Published:** 2022-12-22

**Authors:** Mariana Esther Martinez-Sánchez, José Alberto Choreño-Parra, Elena R. Álvarez-Buylla, Joaquín Zúñiga, Yalbi Itzel Balderas-Martínez

**Affiliations:** 1 Laboratory of Computational Biology, Instituto Nacional de Enfermedades Respiratorias Ismael Cosío Villegas, Mexico City CP 14080, Mexico; 2 Laboratory of Immunobiology and Genetics, Instituto Nacional de Enfermedades Respiratorias Ismael Cosío Villegas, Mexico City CP 14080, Mexico; 3 Instituto de Ecología, Universidad Nacional Autónoma de Mexico, Mexico City CP 04510, Mexico; 4 Centro de Ciencias de la Complejidad (C3), Universidad Nacional Autónoma de Mexico, Mexico City CP 04510, Mexico; 5 Tecnológico de Monterrey, School of Medicine and Health Sciences, Mexico City CP 14380, Mexico

**Keywords:** COVID-19, Boolean model, regulatory model, simulation study, CD4+ T cell, immune response, cytokines, Th1, Th17, Treg

## Abstract

In this model we use a dynamic and multistable Boolean regulatory network to provide a mechanistic explanation of the lymphopenia and dysregulation of CD4+ T cell subsets in COVID-19 and provide therapeutic targets. Using a previous model, the cytokine micro-environments found in mild, moderate, and severe COVID-19 with and without TGF-β and IL-10 was we simulated. It shows that as the severity of the disease increases, the number of antiviral Th1 cells decreases, while the the number of Th1-like regulatory and exhausted cells and the proportion between Th1 and Th1R cells increases. The addition of the regulatory cytokines TFG-β and IL-10 makes the Th1 attractor unstable and favors the Th17 and regulatory subsets. This is associated with the contradictory signals in the micro-environment that activate SOCS proteins that block the signaling pathways. Furthermore, it determined four possible therapeutic targets that increase the Th1 compartment in severe COVID-19: the activation of the IFN-γ pathway, or the inhibition of TGF-β or IL-10 pathways or SOCS1 protein; from these, inhibiting SOCS1 has the lowest number of predicted collateral effects. Finally, a tool is provided that allows simulations of specific cytokine environments and predictions of CD4 T cell subsets and possible interventions, as well as associated secondary effects.

## 1. Introduction

The SARS-CoV-2 virus is the causative agent of the COVID-19 disease, which is responsible for the current global pandemic; however, the severity and etiology of the disease depends on the patient’s immunological response. From an immunological standpoint, COVID-19 is characterized by immune dysregulation. Patients with severe COVID-19 present a cytokine storm and lymphopenia at the same time. The cytokine storm, an uncontrolled systemic inflammatory response mediated mainly by the innate immune system, causes severe immune damage to the patient [1,2], while lymphopenia with a marked reduction in T lymphocytes compromises a successful antiviral response [3,4,5].

COVID-19 patients can be categorized into three groups: mild patients without evidence of pneumonia or hypoxia, moderate COVID-19 patients that present clinical signs of pneumonia but do not require mechanical ventilation, and severe COVID-19 patients that require invasive mechanical ventilation and admission to the intensive care unit [6,7]. Patients with mild symptoms present elevated levels of interleukin (IL)-2 and IL-12 in peripheral blood, moderate patients present an elevated level of interferon (IFN)-γ and IL-12, and severe COVID-19 patients present elevated levels of IL-6, IL-1β, IFN-α, IFN-γ, IL-12, IL-18, IL-33, IL-7, IL-8, IL-2, IL-4, IL-10, transforming growth factor (TGF)-β, TNF-α, CXCL-10/IP-10, granulocyte-colony-stimulating factor (G-CSF), monocyte chemoattractant protein 1 (MCP1), and macrophage inflammatory protein (MIP)1-α [4,5,6,7,8,9,10,11]. However, it is worth noting that the specific levels of cytokines vary depending on the patient, tissue, and local micro-environment [12,13].

CD4+ T (Th) cells play a key role in the activation and inhibition of other immune cells. Naive CD4+ T cells are activated into Th0 cells when they recognize an antigen in a secondary lymphoid organ [14,15]. Depending on the cytokine milieu and other signals from their micro-environment, CD4+ T cells differentiate and expand into distinct effectors (Th1, Th2, Th17) and regulatory (Treg-, Tr1-, Th3-, Th1-like, and Th2-like regulatory cells) subsets. The dynamic equilibrium between effector and regulatory cells is important for infection clearance and to avoid damage to the patient by its immune system [16,17,18,19].

Each CD4+ T cell type is associated with specific cytokines, receptors, transcription factors, and functions [16,19,20,21,22,23,24,25,26,27,28,29,30,31,32,33,34,35,35]. Differentiation between subsets is regulated at the transcriptional level by inhibitions among the different transcription factors and, at the cytokine signaling pathway level, by SOCS proteins [36,37].

CD4+ T cells can experience exhaustion or anergy in response to under- or over-activation of the TCR, where they express inhibitory receptors, produce fewer cytokines, do not proliferate, and may enter apoptosis. These mechanisms are related to avoiding autoimmunity, chronic infections, and cancer. The mechanisms and signaling pathways, as well as the relation of such alterations with different CD4+ T cell subsets, are not understood [38,39,40,41,42].

CD4+ T cells are strongly affected during COVID-19: their count in peripheral blood diminishes, circulation is affected, and there are changes in CD4+ T subset profiles and these cells present over-activation, exhaustion, and altered apoptosis [3,5,43]. Patients with severe COVID-19 have a lower level of Th1 cells and a higher level of Th2 and Th17 cells [44,45]. In these patients, there is an increase in IL-10; however, there seems to be a high variability in the number of regulatory T cells among patients. However, there is no correlation between T cell subsets and IL-10 or the number of Tregs and patient outcome [46,47,48,49,50,51]. Most treatments have focused on blocking specific cytokines, mainly IL-6, or limiting T cell exhaustion [3]. However, we still lack a complete understanding of how these abnormal CD4+ T cell profiles emerge in response to the paradoxical differentiation signals that characterize the cytokine storm associated with severe COVID-19.

Regulatory networks have been shown to provide mechanistic explanations to cell differentiation processes associated with the complex interactions of molecular components. Such models have provided useful bridges between the molecular regulation of a cell and its phenotype in both plant and animal systems [52,53,54]. Indeed, such types of models have provided systemic mechanistic models of cell differentiation in hematopoietic cells, [55,56,57] including CD4+ T cells [58,59,60,61,62]. Some of these models have not been able to integrate regulatory networks with cytokines and signaling pathways to predict differentiation and plasticity in response to the signals in the cells’ micro-environments. The Boolean formalism allows to integrate the regulatory structure of the complex networks. Such structures, rather than the quantitative details of the kinetic interaction functions, seem to be key for the stable states of the dynamic systems involved; therefore, such complex networks can be studied with a few parameters [52,53,54].

Previous Boolean regulatory network models of CD4+ T differentiation on plasticity [58,59,60,61,62] are used and expanded in this study to test cell differentiation under contrasting cytokine micro-environments. The model recovers the naive, Th1, Th2, Th17, Treg, Foxp3(-)I regulatory (Tr), Th1-like regulatory (Th1R), Th2-like regulatory (Th2R), and exhausted (Tex) T cell subsets. In this study, we used the model to simulate the effect of various cytokine micro-environments characterized in mild, moderate, and severe COVID-19 and explore how these affect the differentiation and plasticity of CD4+ T cells. Interestingly, our simulations show that as the severity of the disease increases, the basin size and stability of Th1 cells decrease, while the basin size and stability of Th1R and Tex cells increase. When TGF-β and IL-10 are added to the environment, the Th1 attractor becomes unstable, and Th17 and regulatory subsets are favored. We also used the model to find possible therapeutic interventions that favor the Th1 subtype: activation of the IFN-γ pathway, blockage of the IL-10 or TGF-β pathways, and inhibition of SOCS1.

The most important finding of this approach is that most cytokine signatures prevalent among hospitalized COVID-19 patients interfere with or do not favor the establishment of the Th1 responses required to clear the infection. The model also shows that the contradictory signals in the micro-environments associated with severe COVID-19 favor the Tex, Th1R, and Th17 subsets, increasing the basin sizes, stability, and transitions towards these cell types. In CoV-mild and CoV-mod, which can be classified as pro-Th1 micro-environments, the subset with the bigger basin size and stability is Th1, but Th1R cells are also promoted. In addition, CoV-mild is the only micro-environment with Treg cells. This balanced profile might be beneficial to the host for combating the infection at the minimum tissue damage cost. Conversely, in CoV-sev, the subset with the bigger basin size and stability is Tex. This behavior is probably a result of the presence of contradictory signals in the micro-environment, as in severe COVID-19 there is a high number of Tex attractors that have all SOCS proteins active, which might subsequently hinder each other.

## 2. Materials and Methods

We constructed the CD4+ T regulatory network according to previous models based on experimental information [58,59,60,61,62]. The dynamical analysis of the network was performed using the packages BoolNet [63] and BoolNetPerturb [61].

**Figure 1:**
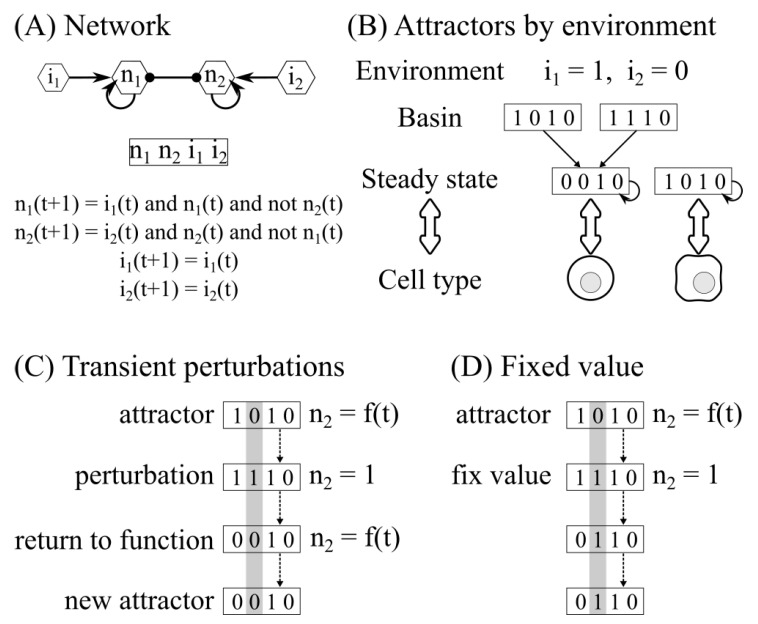
Analysis of the Boolean regulatory network. (**A**) Each node has a value resulting from a Boolean function of its regulators. (**B**) The attractors of the system correspond to the cell types and depend on the micro-environment. (**C**) During transient perturbations, the value of an attractor node is perturbed for a one-time step. (**D**) During transient perturbations, the value of an attractor node is fixed for the whole simulation.

A Boolean network consists of nodes representing molecular components (for example, cytokines, signaling pathways, transcription factors) and edges representing their interactions. The value of the nodes is a discrete variable: 1 if the node is functional and 0 if it is not functional. The value of a node $x_{i}$ at the time t+1 is a function of the value of its regulators at time *t*, according to a logical function that recapitulates available biological information. The state of the network at time *t* is given by the values of all its nodes and will change through time as the regulatory functions are evaluated (Figure 1A).

Eventually, the system will arrive at an attractor corresponding to a cell type. These attractors can be steady states when $x_{t}=x_{t+1}$. The states that lead to the attractor correspond to the basin of attraction. The steady state corresponds to the cell type [52,53,54]. We classified cells depending on the expression of both the characteristic transcription factor and cytokine. Synchronous updating was used in all simulations. To determine the effect of the micro-environment, we first determined the cytokines present in each polarizing environment. Then, we fixed the value of the input nodes according to the cytokines present or absent in that environment, and then determined the attractors (Figure 1B).

Biological systems are subjected to noise, for example, local fluctuations in the cytokine gradient, stochastic effects in signaling pathways, or the binding of transcription factors. In this study, we subjected the system attractors that correspond to cell types to small transient perturbations in the value of the nodes. To do this, we selected a target attractor and node, then we changed the value of the node for a one-time step (bitflip), which creates a new state at Hamming distance one. Then, the perturbation was relaxed, and the new state was updated using the rules of the network until the system reached an attractor and labeled its cell type, which may be the same or different from the original attractor. To study how the micro-environment affects stability and plasticity, we took all possible combinations of attractors and nodes in a micro-environment and modified for one time step the value of the node, then the perturbation was relaxed, and the resulting attractor was determined [61] (Figure 1C).

To study possible therapeutic approaches, we considered an intervention that lasted as long as the infection. We took an attractor or cell type and a target node to simulate this. Then, we fixed the value of the node to zero or one to simulate the effect of the drug, obtained the resulting attractor, and labeled its cell type. We did this for all possible combinations of attractors and nodes in every micro-environment (Figure 1D).

## 3. Results

### 3.1. CD4+ T Cell Regulatory Network

**Figure 2:**
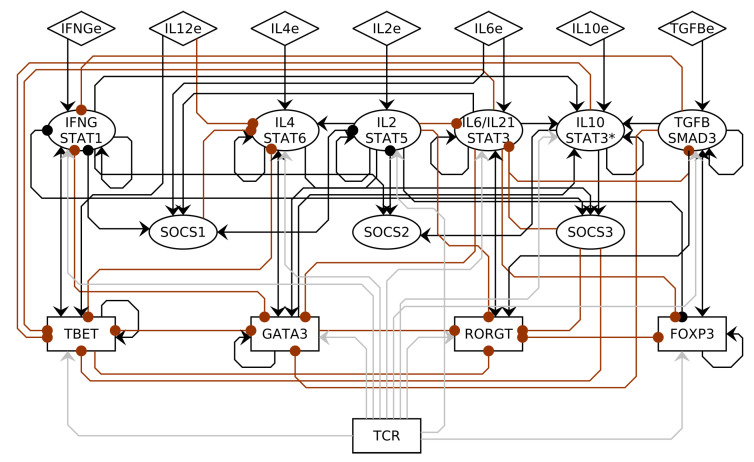
CD4+ T cell regulatory network. The network includes 22 nodes and 80 interactions that can be classified as inputs (diamonds); cytokine signaling pathways, including SOCS proteins (ellipses); and transcription factors (rectangles). Activations are represented with a black or gray arrow, inhibitions are represented with a red dot.

The CD4+ T cell regulatory network was constructed using previous models based on experimental evidence [58,59,60,61,62]. The network includes 22 nodes and 80 interactions (Figure 2, Table S1). The nodes can be classified as inputs, cytokine signaling pathways, SOCS proteins that regulate the signaling pathways, and transcription factors. The input nodes correspond to the extrinsic cytokines in the micro-environment produced by other cells and a node to represent the TCR and co-stimulators signaling cascade. The cytokine signaling pathway nodes correspond to the activation of a cytokine pathway, and this requires the cytokine—produced by another cell (lymphocytes, macrophage, neutrophils, epithelial cells, etc.) or by the same lymphocyte—to bind the receptor, which activate the phosphorylation of a downstream molecule (for example a STAT) that translocate to the nucleus. SOCS proteins regulate the phosphorylation of STAT proteins, modulating the signaling pathways. The transcription factors correspond to necessary key transcription factors; however, they are not sufficient to induce the differentiation of CD4+ T cells into different subsets. Using this information, we constructed the Boolean functions (Table S2).

**Figure 3:**
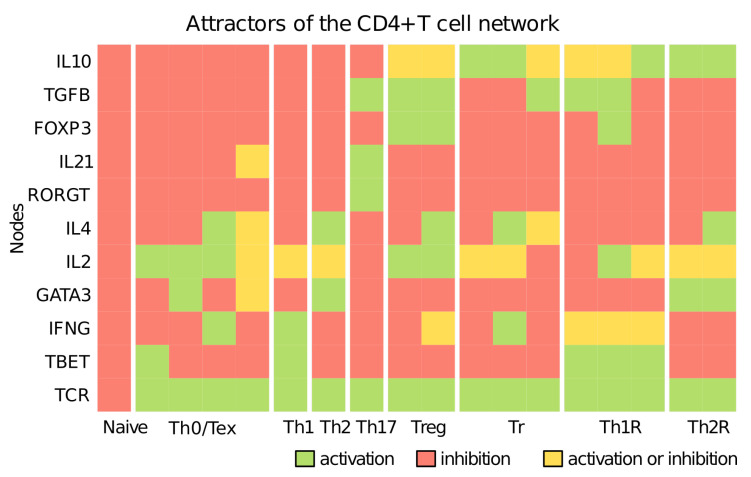
Attractors of the Boolean model to CD4+ T cell subsets. Each row shows the value of one of the key nodes and each column represents an attractor. Attractors that correspond to the same cell type are grouped. Cell types are separated by white spaces. Each node can be active (green) or inactive (red), or both (yellow). The network recovers the attractors corresponding to naive, Th1, Th2, Th17, Treg, FOXP3- regulatory (Tr), Th1-like regulatory (Th1R), Th2-like regulatory (Th2R), Th0, and Tex cells.

The network recovers the attractors that correspond to naive, Th0, Th1, Th2, Th17, Treg, FOXP3- regulatory (Tr), Th1-like regulatory (Th1R), Th2-like regulatory (Th2R), and anergic T cell subsets (Figure 3). Naive T cells have not passed through antigen presentation (TCR = 0) [16,18]. Naive cells are activated by an antigen-presenting cell and differentiate into Th0 cells, which are activated and may express clonal expansion but have not differentiated into a subset [38,39,40,41,42]. Effector cells activate other cells of the immune system: Th1 cells express T-bet and IFN-γ, Th2 cells express GATA-3 and IL-4, and Th17 cells express, IL-6, IL17A, and IL-17F [16,19,20,21,22]. Regulatory cells modulate the immune system: Treg cells express Foxp3 and TGF-β and may express IFN-γ [23,24,25,26]; Tr cells do not express Foxp3 but produce IL-10 and/or TGF-β, which correspond to Tr1, Th3 or IL-10+TGF-β+ cells [27,28,29,30]; Th1-like regulatory T cells (Th1R) express T-bet and Foxp3 [31,32,33,34]; TGF-β or IL-10 and Th2-like regulatory T cells (Th2R) express GATA-3 and IL-10 [31,34,35,64]. Th cells may become exhausted if the TCR is overstimulated, where they express no cytokines or transcription factors [38,39,40,41,42]. Th0/Tex cells can correspond to two states, namely Th0 cells that have not experienced differentiation and anergic or exhausted T cells; however, it is not possible to distinguish these phenotypes because the model lacks marker molecules and some of the signaling pathways are still open to question [38,39,40,41,42]. In general, we considered that a cell with TCR = 1 that could not be classified as an effector or regulatory cell was a Th0 cell at the beginning of the simulation, and it was a Tex cell if the state was reached at the end of the simulation as a result of a perturbation to an effector or regulatory attractor.

### 3.2. The Severity of COVID-19 Affects the Differentiation of CD4+ T Cells

CD4+ T cell differentiation is heavily dependent on the micro-environment. This is especially important during an active infection, where the signals produced by the infected cells modulate the differentiation of CD4+ T cells, and these cells activate or inhibit other cells of the immune system. We model the cellular micro-environment with input nodes that represent the extrinsic cytokines produced by other immune system cells. Given that the model is Boolean, the extrinsic cytokines can be present at a low (0) or high (1) level. The local concentration of cytokines can vary depending on the patient, the cytokines produced by the cells in the local micro-environment, and the cytokines produced by the same CD4+ T cell [12,13]. Using available information [4,5,6,7,8,9,10,11], we defined five micro-environments for analysis (Table S3): mild COVID-19 (CoV-mild) with high levels of IL2e and IL12e; moderate COVID-19 (CoV-mod) with high levels of IFNGe and IL12e; severe COVID-19 (CoV-sev) with high levels IFNGe, IL-12, IL4e, and IL6e; severe COVID-19 with extrinsic TGF-β (CoV-TGFB) with IFNGe, IL-12, IL4e, IL6e, and TGFBe; and severe COVID-19 with extrinsic IL-10 (CoV-IL10) with IFNGe, IL-12, IL4e, IL6e, and IL10e.

**Figure 4:**
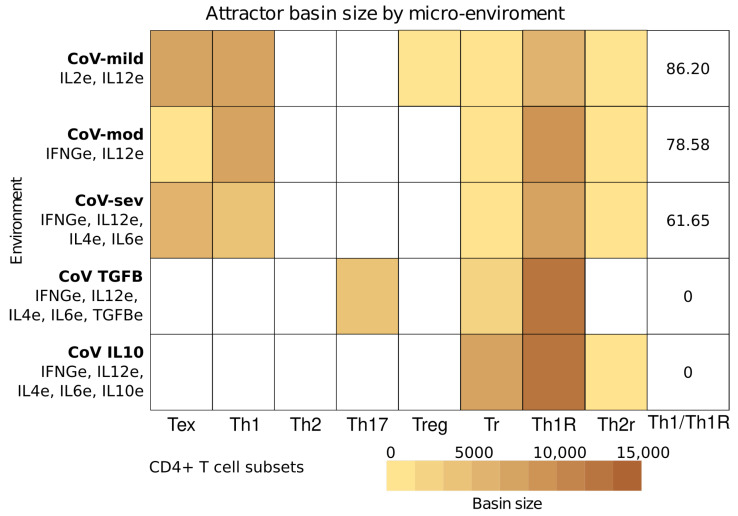
Subsets recovered by the model depend on the inputs, which represent the extrinsic cytokines in the micro-environment. Each row corresponds to a micro-environment, while the column represents the corresponding subset or attractor. The color of a cell depends on the basin of attraction, the number of states that converge to the attractor.

Using these micro-environments, we simulated the response of CD4+ T cells to various micro-environments (Figure 4). CoV-mild, CoV-mod, and CoV-sev simulations recovered highly similar CD4+ T cell profiles: Th1, Tr, Th1R, Th2R, and Th0/Tex subsets in the three environments with the addition of Treg in CoV-mild, which are in accordance with the observed heterogeneity of effector and regulatory cells in vivo [4,5,6,7,8,9,10,11,46,65]. However, the basins of attraction, the number of states that lead to an attractor, change between the environments. In CoV-sev, the Tex basin is four times bigger than in CoV-mod, while the Th1 basin is half as big, with few changes in regulatory cell types. In CoV-TGFB and CoV-IL10, the CD4+ T cell differentiation profile changes radically. In CoV-TGFB, the model recovers attractors that correspond to Th17, Th1R, and Tr, while in CoV-IL10, the model recovers the Th1R, Th2R, and Tr attractors with no Treg or Th1 attractors [9,11,46,65]. Th1R cells, which inhibit the Th1 response [66], are the most common regulatory cells in the three micro-environments. There is also an important number of Th2R and IL-4+IL10+ Tr cells that could explain both the increase of Th2 cells and IL4 and IL10 in severe COVID-19. Furthermore, as the severity of the disease increases, the balance of Th1 and Th1R cells shifts in favor of the regulatory cells, and there are no Th1 attractors in CoV-TGFB and CoV-IL10, which implies an imbalance between the antiviral Th1 response and the Th1R regulatory response.

### 3.3. Severe COVID-19 Decreases the Stability of Th1 Cells and Increases the Transitions towards Tex and Th17 Subsets

The concentration of cytokines, signaling pathways and gene expression of cells are all subject to stochastic noise, which can affect the expression profile of the cell. To study the stability of a CD4+ T cell, we took an attractor and transiently perturbed one of its nodes for a single time step and then determined if the simulated cell returned to an attractor corresponding to the same cell type or another. We repeated this experiment for every node in every attractor.

**Figure 5:**
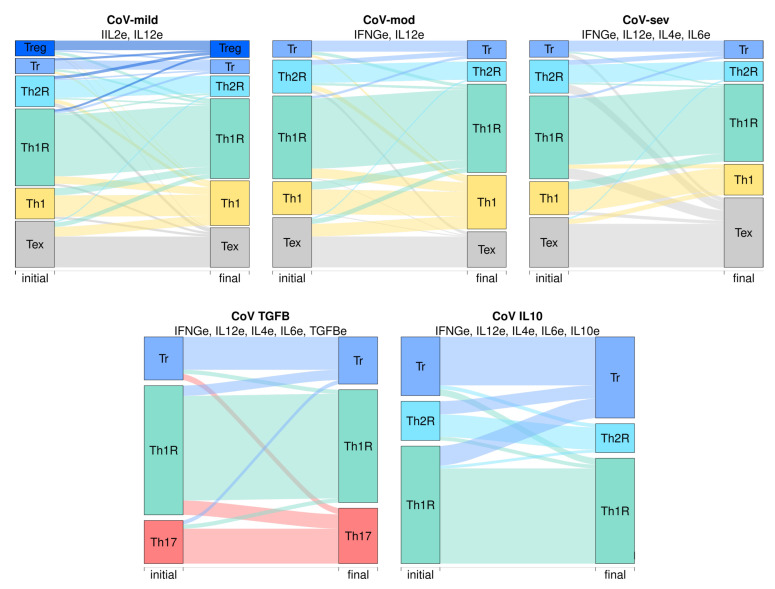
CD4+ T cells stability and transitions in response to the micro-environment. For each environment, we calculated the attractors; then, for each attractor, we transiently perturbed every node independently for one time step to determine the stability of the different cell types. Each stability experiment is represented by a flux diagram, where the colored boxes correspond to each cell type. The initial state is on the left of the diagram and the final state is on the right. The height of the bar corresponds to the basin size of the attractor. The width of the lines between boxes represents the transitions between attractors.

The model recovers Th1, Th1R, Th2R, Tr, and Th0/Tex attractors in CoV-mild, CoV-mod, and CoV-sev micro-environments, with a Treg addition in CoV-mild (Figure 5). The Th0/Tex subset is composed of attractors that only express a transcription factor or a non-regulatory cytokine but not both, because they cannot be classified as an effector or as regulatory cells. At the beginning of the simulation, these cells can be considered Th0 cells, as the TCR has been activated; however they have not differentiated into a given subset because they lack extrinsic signals or because the contradictory signals have blocked each other. However, after some time, the cells in this compartment can be classified as Tex, especially considering that some of them come from differentiated subsets, such as Th1 or regulatory cells, but their expression patterns are altered after being subjected to a transient perturbation.

The stability of the recovered subsets changes depending on the cytokine milieu. In CoV-mild and CoV-mod, Th1 and Th1R are the most stable subsets, and most transitions between subsets are towards Th1 with a significant number of transitions towards Treg in CoV-mild. In CoV-mod, Th1 and Th1R are the most stable subsets, and most transitions between subsets are towards Th1. However, in CoV-sev, Tex and Th1R are the most stable subsets, and most transitions between the subsets are towards Tex. In CoV-TGFB, Th17 is the most stable subset, and most transitions between subsets are towards it, while Th1R is the least stable. In CoV-IL10, Tr is the most stable subset, and most transitions between subsets are towards it, while Th1R is the least stable. The increase in the basin size, stability, and transitions towards the Tex attractor in CoV-sev seems to be associated with the competition between contradictory signals in CD4+ T cells, as there was an increase of anergic attractors with active SOCS nodes. With time, the sharp change in the stability of the Th1, Th17, and Tex subsets between CoV-mod and CoV-sev could strongly impact the CD4+ T cell populations. In severe COVID-19, the number of cells in the Th1 compartment will decrease, and the number of Tex cells will increase in response to the transient perturbation caused by small variations in the cytokines in the micro-environment, the activation of signaling pathways, or the expression of transcription factors. This observation could explain the increase in exhausted or abnormally activated CD4+ T cells in severe COVID-19.

### 3.4. IFNG, TGF-β, and SOCS1 Are Critical for the Immunomodulation of COVID-19

Modulation of CD4+ T cell subsets could improve patient outcome in COVID-19, not only by activating the adaptive antiviral immune response, but also, by modulating the aggressive innate inflammatory response. Most treatments have focused on blocking IL-6 or limiting T cell exhaustion by modulating the TCR and co-factors signaling. However, the differentiation of the CD4+ T cell subsets depends strongly on the cytokine milieu. Interventions can be classified depending on the type of node targeted (extrinsic cytokine, signaling pathway, SOCS protein, or transcription factor) or whether they constitutively activate or inhibit a specific node. For example, treatment with a monoclonal antibody against the IL-6 receptor (for example the drug tocilizumab) corresponds to the intervention IL6R = 0. We include a supplementary table (Table S4) with drugs that may correspond to the theoretical interventions constructed using publicly available drug databases; however, the pertinence of these interventions should be carefully analyzed by clinicians [67,68].

To propose possible treatments that complement existing interventions, we fixed the values of the nodes from the system and determined how they affected the differentiation and plasticity of CD4+ T cells (Table 1). The intervention lasts the whole run (t=∞) to simulate the treatment of a patient until the infection is resolved. We focused on the interventions that induced transitions towards Th1 in the severe COVID-19 micro-environments (CoV-sev, CoV-TGFB, CoV-IL10). To study the possible secondary effects, we determined the effect of the intervention on other micro-environments and cell types.

None of the interventions worked for all micro-environments, and all had secondary effects. We found four permanent interventions that induced transitions from Tex or Th1R cells towards Th1: IFNG = 1, IL10 = 0, TGFB = 0, and SOCS1 = 0. All of these interventions imply modulating the signaling pathways or SOCS proteins. Given that this intervention strongly affects the differentiation program, especially those that target transcription factors, they can cause the appearance of hybrid subsets, such as Th17-like regulatory cells.

Treatments may be interrupted or subject to other stochastic events. We simulated the interventions lasting for a limited number of time steps and then removed the intervention to model these situations. We show the results for *t* = 1 given that most time lengths had the same effect (Table 1). Activating the IFN-γ pathway caused transitions from Tex to Th1 in CoV-sev, but its effect was limited in the presence of TGFBe or IL10e, and its secondary effects included multiple transitions towards Th1R, which could limit the beneficial effect of the intervention. Inhibiting the IL-10 or the TGF-β pathway caused transitions from Th1R to Th1, but it also caused several transitions towards Tex and Th17, which means that careful consideration should be taken into account before using these interventions. Interestingly, the inhibition of SOCS1 caused the transition of Tex towards Th1 cells, with only the secondary effect of Th17 → Th1R in the CoV-TGFB environment. This intervention seems to work by reducing the deadlock between competing signaling pathways. The transient interventions IL6 = 1 and SOCS3 = 0 also caused a transition from Th1R to Th1.

Given that the effect of the intervention depends heavily on the specific cytokine profile of the patient, we included a small application to simulate the different cytokine micro-environments and show all the possible transitions in that micro-environment: https://yalbibalderas.shinyapps.io/cd4tcell_covid19_app/ (accessed on 13 December 2022).

## 4. Discussion

In this paper, we present a model to explain the behavior of T CD4+ cells during COVID-19 and to propose possible interventions to modulate these cells. The most important finding of this approach is that most cytokine signatures prevalent among hospitalized COVID-19 patients interfere or do not favor the establishment of the Th1 responses required to clear the infection. Remarkably, the model also shows that the contradictory signals in the micro-environments associated with severe COVID-19 favor the Tex, Th1R, and Th17 subsets, increasing the basin sizes, stability, and transitions towards these cell types. In CoV-mild and CoV-mod, which can be classified as pro-Th1 micro-environments, the subset with the bigger basin size and stability is Th1; however, Th1R cells are also promoted. In addition, CoV-mild is the only micro-environment with Treg cells. Conversely, in CoV-sev, the subset with the bigger basin size and stability is Tex. This behavior is probably a result of the presence of contradictory signals in the micro-environment, as in severe COVID-19 there is a high number of Tex attractors, that have all SOCS proteins active, which might subsequently hinder each other.

Importantly, all the CD4+ T cells subsets enriched in severe COVID-19 alter the Th1-induced antiviral defenses. The Tex cells in the model correspond mainly to hyper-activated cells that are unresponsive by contradictory cytokines signals that can block the differentiation or “exhaust” a previously differentiated cell. Meanwhile, Th1R cells merit special attention as they express Tbet and a combination of IFNγ, Foxp3, TGF-β, and IL-10, which correspond to previously reported subsets. These cells have a role in the modulation of the Th1 response, as they express similar chemokine receptors [66,69]. This attractor was commonly found in the simulations with a high basin size and stability in severe COVID-19. Furthermore, the proportion of Th1/Th1R cells increased with the severity of the disease, and while a number of Th1R and other regulatory cells may be beneficial to avoid immune damage, the increase in Th1R cells could be associated with an over-regulated antiviral response. This could hint at Th1-like regulatory cells as part of the immune dysregulation, where the host is unable to mount an effective antiviral response. The increase in the number of Th1R cells dampers Th1 responses, while not protecting the host completely from the damage caused other immune cells like macrophages or neutrophils. Finally, Th17 cells promote neutrophilic inflammation, causing extensive tissue damage and organ failure. As such, increased neutrophils in the circulation have been recently considered as a readout of severe COVID-19 [70].

Our model also predicts that IL-10 and TGF-β have a strong effect on the system, especially in conjunction with the CoV-sev micro-environment. In the presence of TGFBe, Th1 differentiation is inhibited and Th17 emerges as the most stable subset, while IL10e favors the Tr subset and inhibits all regulatory characteristics. Notwithstanding, these results should be taken with care, as the effect of TGF-β in CD4+ T cell differentiation is concentration-dependent. Moreover, the CoV-sev, CoV-TGFB, and CoV-IL10 states could coexist in a single patient depending on the local concentration of TGF-β and IL-10; creating a systemic mix with few Th1 cells that induce an antiviral response, Th17 cells that activate neutrophils and macrophages, a mix of regulatory cells (Tr, Th1R, Th2R), and a high level of Tex cells. Given that Th1R cells are the more common regulatory cells in the three micro-environments, these subsets could have a crucial systemic role in inhibiting the Th1 responses while failing to modulate other types of the inflammatory response, which correlates with the immune damage characteristic of COVID-19, as mentioned above. Taking together, our results imply that the few numbers of Th1 cells and a high number of exhausted, abnormally activated and apoptotic T cells in COVID-19, is not only the result of an abnormal TCR activation, but also of problems in the differentiation caused by the abnormal immune response in severe COVID-19.

Our findings might also influence the views of researchers regarding future applications in immunotherapeutics against COVID-19 morbidity and mortality. To date, treatments with the objective to improve the quantity and quality of CD4+ T cells in COVID-19 have focused on addressing hyperactivation, anergy, or modulating specific cytokines, such as IL-1 and IL6 [43,71]. In this regard, our model projects at least four possible interventions to promote a protective Th1 response: the activation of the IFN-γ pathway, blockage of the IL-10 or TGF-β pathways, and inhibition of SOCS1. All these interventions proved to be effective in promoting a transition towards the Th1 attractor; however, their secondary effects should be considered, as they can increase the Tex, Th1R, and Th17 responses. It is important to take into account the balance between Th1 and Th1R responses, as completely blocking the Th1R response could cause further immune damage. For example, the blockage of the IL-10 or TGF-β pathways caused the transition of Th1R cells towards Tex cells. Given the specificity and consequences of the different treatments, we recommend further studies into the inhibition of SOCS1 [72], as this intervention favors the transition from Tex towards Th1 with the transition of Th17 to Th1R cells, both of which have positive effects for patients. In the first three cases, there exist investigational or approved drugs that correspond to the intervention (Table S4), while in the case of SOS1, there are experimental peptides under development [72], which could be evaluated by clinicians.

CD4+ T cell populations are heterogeneous both in silico and in vivo. Our model recovers effector subsets, such as Th1 and Th17, and various regulatory subsets in every environment, such as Tr, Th1R, and Th2R. Available information about the regulatory subsets is limited. Distinguishing between Th1, Th1-like regulatory cells, and Tregs cells can be complicated; however, taking into account the balance between effector and regulatory cells could increase the antiviral response while modulating the immune damage, which is critical in COVID-19. Many experimental studies (including those that use commonly sold cytokine panels) focus only on effector cytokines or transcription factors but not on regulatory cytokines, which can complicate classifying regulatory and hybrid CD4+ T cells. This could mean that, for example, some cells classified as Th2 cells by an experimental study could be both IL4+ and IL10+ and be classified as Th2R or Tr by our model.

A strong limitation of this model is that it fails to capture the dynamics of the TCR and TNF-α. This is a result of both the Boolean approach and the network construction. The Boolean approach combines a binarization of concentrations, mathematical simplification of pathways, and analysis in the steady state, which are lost with the simplification or require temporal delays, with nodes that simulate complex formation or continuous approaches to recover the expected behavior [62,73]. Further models should use non-simplified ordinary differential equations to capture the effect of the signal and temporal dynamics. Key cytokines such as IL-2 and TNF-α modulate the TCR signaling pathway, and the TCR modulates the differentiation into subsets; however, these interactions are not included in the model [74,75,76,77].

Another limitation is that we cannot clearly differentiate the Th0 and Tex attractors. The Tex state, which corresponds to anergy, exhaustion, and senescence, is a functional state, and markers are shared between these states and vary between subsets, especially when taking into account the various regulatory cells, and we still lack a clear understanding of its signaling pathways and regulation [38,39,42,78]. Developing an integrative model that includes both the TCR and the differentiation of CD4+ T cells and that is able to recover the activation, effector, regulatory, anergic, exhausted, and apoptotic processes is still an open challenge; furthermore, while the model describes the basin size and plasticity of CD4+ T cells, this is not necessarily a measure of the population behavior, and while the basin of attraction can be considered a rough estimate of the population size [79], the initial population and the effect of stochastic noise will affect the proportion of the populations through time.

This study opens multiple paths for further theoretical and experimental research. This methodology could prove useful to understand the behavior and suggest therapeutic targets for other key cells of the immune system, like macrophages and neutrophils in COVID-19 and other diseases, and auto-immune conditions. Furthermore, it opens multiple questions of how the populations of immune cells will evolve through time in circumstances where they are subjected to both stochastic noise and directed clinical interventions. On the experimental side, the model proposes several possible interventions, which can be achieved with approved or investigational drugs (Table S4) [67,68]. In the case of the SOCS1 = 0 intervention, there exists experimental peptides that require further research [72]. However, while these treatments may improve the differentiation of CD4+ T cells, it will probably be necessary to use them in conjunction with treatments that modulate the TCR response and consider the possible effects on other cell types.

Strikingly, our model also highlights the need for obtaining additional information about the immune profiles of candidate patients before administering immunotherapeutics, since the effects of each proposed intervention might vary in different micro-environments. This is in contrast with current practices in clinical settings, where the administration of an immunotherapeutic agent, such as tocilizumab, to severe COVID-19 patients is guided by measurements only of IL-6 concentrations. In particular, we urge clinicians to consider regulatory cytokines, such as IL-10 and TGF-β, which have a strong effect on the immune profile and are not always measured. The most adequate treatment should be selected according to the individual cytokine and immune cell subset of each patient, and other adjuvant interventions should be considered to avoid possible secondary effects. Models such as the one presented here can integrate available cytokine measures and predict both the CD4+ T subsets and the secondary effects of interventions in a personalized manner.

## Figures and Tables

**Table 1:** Interventions in severe COVID-19 micro-environments that induce transitions towards Th1 cells and their secondary effects.

Environment	CoV-sev		CoV-TGFB		CoV-IL10	
Perturb Time	*t* = *∞*	*t* = 1	*t* = *∞*	*t* = 1	*t* = *∞*	*t* = 1
IFNG = 1	Tex → Th1	Tex → Th1	-	-	-	-
	Th2R → Th1R, Tr → Th1R	Th2R → Th1R, Tr → Th1R	Th17 → Th1R, Tr → Th1R	Th17 → Th1R, Tr → Th1R	Th2R → Th1R, Tr → Th1R	Th2R → Th1R, Tr → Th1R
IL10 = 0	Th1R → Th1	Th1R → Th1	-	-	Th1R → Th1	-
	Th1R → Tex, Th2R → Tex, Tr → Tex	Th1R → Tex, Th2R → Tex, Tr → Tex	Th1R → Th17, Tr → Th17	Th1R → Th17, Tr → Th17	Th1R → Tex, Th2R → Tex, Tr → Tex	Th1R → Tr, Th2R → Tr, Tr → Th1R
TGFB = 0	Th1R → Th1	Th1R → Th1	Th1R → Th1	-	-	-
	Th1R → Tex, Tr → Tex	Th1R → Tex, Tr → Tex	Th17 → Tex, Th1R → Tex, Tr → Tex	Th1R → Th17, Tr → Th17	-	-
SOCS1 = 0	Tex → Th1	Tex → Th1	-	-	-	-
	-	-	Th17 → Th1R	Th17 → Th1R	-	-
IL6 = 1	-	Th1R → Th1	-	-	-	-
	Th1 → Tex, Th1R → Tex, Th1R → Tr, Th2R → Tr	Th1R → Tex, Th2R → Tex, Tr → Tex	Th1R → Tr	Th1R → Tr	Th1R → Tr, Th2R → Tr	Th1R → Tr, Th2R → Tr
SOCS3 = 0	-	Th1R → Th1	-	-	-	-
	Th1 → Tex, Th1R → Tex, Th1R → Tr, Th2R → Tr	Th1R → Tex, Th2R → Tex, Tr → Tex	Th1R → Th17, Th1R → Th17R, Tr → Th17R	Th1R → Tr	Th1R → Tr, Th2R → Tr	Th1R → Tr, Th2R → Tr

## Data Availability

All data, scripts, resulting tables, and figures can be found at: https://github.com/Laboratorio-de-Biologia-Computacional/CD4Tcell_COVID19 and https://yalbibalderas.shinyapps.io/cd4tcell_covid19_app/ (accessed on 13 December 2022).

## Supplementary Materials

The following supporting information can be downloaded at: https://www.mdpi.com/article/10.3390/pathogens12010018/s1, Table S1: Boolean rules of the CD4+ T cell regulatory network; Table S2: Labeling rules for the CD4+ T cell regulatory network; Table S3: Environments used to simulate the CD4+ T cell regulatory network; Table S4: Candidate drugs that correspond to the theoretical interventions.
